# Respective stemness and chondrogenic potential of mesenchymal stem cells isolated from human bone marrow, synovial membrane, and synovial fluid

**DOI:** 10.1186/s13287-020-01786-5

**Published:** 2020-07-25

**Authors:** Paul Neybecker, Christel Henrionnet, Elise Pape, Laurent Grossin, Didier Mainard, Laurent Galois, Damien Loeuille, Pierre Gillet, Astrid Pinzano

**Affiliations:** 1 UMR 7365 CNRS-Université de Lorraine, IMoPA (Ingénierie Moléculaire et Physiopathologie Articulaire), Biopôle de l’Université de Lorraine, Campus Brabois-Santé, 9 Avenue de la Forêt de Haye, BP 20199, F54505 Vandœuvre-Lès-Nancy, France; 2 Laboratoire de Pharmacologie, Toxicologie et Pharmacovigilance, Bâtiment de Biologie Médicale et de Biopathologie, CHRU de Nancy-Brabois, 5 Rue du Morvan, F54511 Vandœuvre-lès-Nancy, France; 3 Service de Chirurgie Orthopédique, Traumatologique et Arthroscopique, CHRU Nancy, 29 avenue du Maréchal de Lattre de Tassigny CO 60034, F54035 Nancy, France; 4 Service de Rhumatologie, CHRU de Nancy, Hôpitaux de Brabois, Bâtiment des Spécialités Médicales, 5 rue du Morvan, F54511 Vandœuvre-lès-Nancy, France; 5 Contrat d’Interface, Service de Rhumatologie, Hôpital de Brabois, Bâtiment Spécialités Médicales, F54511 Vandœuvre lès Nancy, France

**Keywords:** Mesenchymal stromal stem cells, Cartilage engineering, Synovial membrane, Synovial fluid, Bone marrow, Chondrogenic differentiation, Growth factors

## Abstract

**Background:**

MSCs isolated from bone marrow (BM-MSCs) have well-established chondrogenic potential, but MSCs derived from the synovial membrane (SM-MSCs) and synovial fluid (SF-MSCs) are thought to possess superior chondrogenicity. This study aimed to compare the in vitro immunophenotype and trilineage and chondrogenic potential of BM-MSCs to SM-MSCs and SF-MSCs.

**Methods:**

MSCs were isolated from bone marrow (BM-MSCs), synovial membrane (SM-MSCs), and synovial fluid (SF-MSCs) extracted from the hips (BM) and knees (SM and SF) of advanced OA patients undergoing arthroplasty. Flow cytometric analysis was used at P2 to evaluate cell stemness. The trilinear differentiation test was performed at P2. At P3, MSC-seeded collagen sponges were cultured in chondrogenic medium for 28 days. Chondrogenic gene expression was quantified by qRT-PCR. Finally, the implants were stained to assess the deposition of proteoglycans and type II collagen.

**Results:**

Despite variability, the immunophenotyping of BM-MSCs, SM-MSCs, and SF-MSCs was quite similar. All cell types were positive for the expression of stem cell markers and negative for exclusion markers. Additionally, chondrogenic differentiation and hypertrophy were more pronounced in BM-MSCs (*ACAN*, *SOX9*, *COL2B*, and *COL10A*) than in SF-MSCs, with SM-MSCs having intermediate characteristics. Concerning matrix synthesis, the three cell types were equipotent in terms of GAG content, while BM-MSC ECM synthesis of type II collagen was superior.

**Conclusions:**

Chondrogenic MSCs are easily collected from SM and SF in advanced human OA, but in vitro chondrogenesis that is superior to age-matched BM-MSCs should not be expected. However, due to intra-articular priming, SF-MSCs did not overexpress hypertrophic gene.

## Background

As hyaline cartilage lacks vasculature, neurons, and a lymphatic system, it possesses reduced self-healing potential, and as a consequence, focal or diffuse chondral defects lead to osteoarthritis (OA). Regeneration of posttraumatic injured cartilage remains an essential objective in orthopedics. In 1994, autologous chondrocyte implantation (ACI) was introduced in the clinic as a cell therapy for cartilage defects [1, 2]. During the last decade, other cell therapy and tissue engineering techniques have emerged, but most used chondrocytes, which are unique, specialized resident cartilage cells [3, 4]. However, ACI has several disadvantages: (i) development of cicatricial fibrotic cartilage, (ii) cartilage biopsy is invasive with possible donor site morbidity, (iii) the number of resident chondrocytes is low, and (iv) there is rapid dedifferentiation during monolayer cell expansion [5].

Since the discovery of mesenchymal stem cells in bone marrow [6], stem cell therapy has become a very promising and advanced scientific research topic. Stem cells originate from two primary sources: adult body tissues and embryos. Scientists are also working on ways to develop stem cells from other cells using genetic reprogramming techniques. Multipotent stromal cells are heterogeneous and contain several populations, including stem cells. The term MSCs should be used for multipotent mesenchymal stromal cells rather than mesenchymal stem cells, since the cells isolated do not contain a pure population of stem cells. The isolation of MSCs according to current criteria produces heterogeneous, non-clonal cultures of stromal cells containing stem cells with different multipotential properties, committed progenitors, and differentiated cells [7–9]. Concerning cartilage engineering, some adult MSCs have been promoted, such as cartilage, bone marrow (BM), fat, or periosteal mesenchymal stem cells.

The existence of chondroprogenitor cells in synovial tissue was discovered in the 2000s while characterizing synovial chondromatosis, a rheumatological disease characterized by cartilaginous nodule formation inside the synovial cavity [10], and their stemness was confirmed in 2006 [11]. In addition, human multipotent MSCs were isolated from the synovial membrane (SM-MSCs) of knee joints in 2001 [12]. MSCs from synovial fluid (SF-MSCs) were isolated later in 2004. Currently, these cells are of great interest in cartilage engineering due to their easy accessibility with arthrocentesis and their stemness trilineage differentiation, self-renewal capacity, and immunosuppressive properties [13]. In terms of chondrogenesis, both SM-MSCs and SF-MSCs should have the advantage of sharing a common marker with chondrocytes, CD44, which is involved in hyaluronan synthesis [14]. Nevertheless, to the best of our knowledge, a direct comparison of these three cell types (BM-, SM-, and SF-MSCs) is lacking in humans. Various scaffolds, environmental growth factors (especially the TGF-β superfamily) promote SM- and SF-MSC chondrogenic differentiation [15], participating in the heterogeneity of the data.

In this study, we compared the respective stemness and chondrogenicity of 2 intra-articular components of advanced OA stem cells, SM- and SF-MSCs, versus BM-MSCs, which are considered the gold standard. To this end, we first performed flow cytometry analysis and trilineage differentiation. We then investigated their respective chondrogenic differentiation (PCR, histology, and immunohistochemistry) into 3D collagen sponges for 28 days under the influence of TGF-β1 and normoxia.

## Materials and methods

### Isolation and expansion of MSCs derived from human bone marrow, synovial membrane, and synovial fluid

MSCs were isolated from human bone marrow following total hip (BM-MSCs) or knee arthroplasty (SM- and SF-MSCs), both for advanced osteoarthritis (OA), after informed consent and with the approval of our local ethics committee (file DC 2014—2148, authorized July 10, 2014). Each heparinized bone marrow sample was diluted in PBS (phosphate-buffered saline) solution and then centrifuged at 1500 rpm for 5 min. The pellet was resuspended in complete culture medium and then seeded in Petri dishes at 4 × 10^6^ cells/dish at 37 °C in a humidified atmosphere containing 5% (v/v) CO_2_. SM samples were aseptically isolated, finely minced, washed in PBS, digested overnight with collagenase B at 2 mg/ml and 37 °C, and centrifuged at 1500 rpm for 5 min. The pellets were resuspended in complete culture medium and seeded in Petri dishes at 10^6^ cells/dish. Human SF samples were diluted 1:6 in expansion culture medium and plated in 55 cm^2^ Petri dishes.

MSCs derived from various tissues (all unmatched) were expanded in monolayers separately in proliferation medium containing Dulbecco’s modified Eagle’s medium with low glucose (DMEM-LG, Gibco) supplemented with 10% fetal bovine serum (FBS, Sigma), 1 ng/ml basic fibroblast growth factor (bFGF, Miltenyi Biotec), 1% glutamine (Gibco), and 1% penicillin-streptomycin (Gibco). The dishes were cultured at 37 °C with 5% humidified CO_2_. The medium was unchanged for the initial 3 days and then changed twice per week until confluence was reached. The non-adherent cells were discarded through subsequent changes of the medium. When the adherent cells reached approximately 80% confluence, the MSCs were trypsinized (trypsin-EDTA 0.05%, Gibco) and plated at a density of 0.5 × 10^6^ cells/dish. The medium was changed the following day and then every 2–3 days.

Predifferentiation medium was used at the final passage (P3) before seeding the MSCs into collagen sponges. This medium was composed of Dulbecco’s modified Eagle’s medium with high glucose (DMEM-HG, Gibco) supplemented with 10% fetal bovine serum (FBS, Sigma), sodium pyruvate (110 μg/ml, Gibco), bFGF (1 ng/ml, Miltenyi Biotech), 1% penicillin-streptomycin (Gibco), PAD chondrogenic supplements (PAD; proline (40 μg/ml, Sigma), L-ascorbic acid-2-phosphate (50 μg/ml, Sigma)), and dexamethasone (10^–7^ M, Sigma) [16, 17].

### Trilineage differentiation potential of BM-, SM-, and SF-MSCs

#### Chondrogenesis

MSCs were trypsinized at P2 and centrifuged at 300×*g* for 10 min to form pellets (0.5 × 10^6^ cells/pellet). Chondrogenic differentiation was induced by 3D culture for 28 days in a dedicated medium [18]. At D28, the pellets were fixed in 4% paraformaldehyde (PFA, Sigma) for 24 h at 4 °C. Samples were subsequently dehydrated and embedded in paraffin. Five-micron-thick sections were obtained using a microtome (Leica) and stained with Alcian blue to observe the proteoglycan content in the pellets.

#### Adipogenesis

MSCs were trypsinized at P2 and seeded in 24-well plates at 5000 cells/well. Adipogenic MSC differentiation was induced for 21 days with a dedicated medium [18]. On D21, monolayer cells were fixed with 4% PFA for 30 min and were stained with oil red (Sigma) to detect lipid vacuoles.

#### Osteogenesis

At P2, MSCs were seeded in 24-well plates at 10,000 cells/well and cultured in commercialized osteogenic medium (Miltenyi Biotech) for 14 days [18]. At D14, monolayer cell cultures were fixed with 4% paraformaldehyde for 30 min and stained with alizarin red (Sigma) to visualize calcium deposits.

### Flow cytometry analysis of BM-, SM-, and SF-MSCs

At P2, MSCs were rinsed with a blocking solution (0.5% BSA (A-9667 Sigma Aldrich, France) in 1× PBS) and distributed at 500,000 cells/tube. The cells were centrifuged (300×*g*, 5 min), and the pellets were resuspended in an immunoblotting solution containing either different pairs of antibodies [anti-CD45 (BD Pharmingen), anti-CD34 (BD Pharmingen), anti-CD73 (BD Pharmingen), anti-CD90 (Beckman Coulter), or anti-CD105 (Beckman Coulter)] or 100 μL of blocking solution as a negative control. After an incubation for 45 min (4 °C, in the dark), the cells were washed with PBS (Gibco) and centrifuged (300×*g*, 5 min). The pellets were resuspended in 300 μL of PBS and the tubes were analyzed by using a flow cytometer (Gallios, Beckman Coulter).

### Respective chondrogenic potential of BM-, SM-, and SF-MSCs in collagen sponges

The 3D culture was performed by using collagen sponges manufactured by Symatèse Biomatériaux (Chaponost, France) that were composed of 95% type I collagen and 5% type III collagen. The size of each collagen sponge was 5 mm in diameter and 2 mm in thickness. MSCs derived from the three tissue types (BM, SM, and SF) were seeded at P4 into sponges at a density of 0.5 million cells/sponge. The sponges were cultured under normoxia conditions (20% O_2_, v/v) at 37 °C in a humidified atmosphere containing 5% CO_2_ (v/v). Two conditions were tested: (i) control medium without chondrogenic growth factor composed of DMEM-HG supplemented with 1% ITS + premix (BD Biosciences), 1% glutamine (Gibco), sodium pyruvate (110 μg/ml, Gibco), 1% penicillin-streptomycin (Gibco), and PAD chondrogenic supplements (Sigma) as described previously and (ii) chondrogenic medium composed of control medium supplemented with TGF-β1 (10 ng/ml, Miltenyi Biotech). At D28, gene expression, and biochemical, histological, and immunohistochemical analyses were assessed.

### Real-time RT-PCR analysis of MSC-seeded sponges at D28

At D28, the total cell RNA was extracted using a RNeasy mini kit (Qiagen), according to the manufacturer’s instructions. The RNA was quantified spectrophotometrically and reverse transcribed with an iScriptcDNA Synthesis Kit (Bio-Rad) according to the manufacturer’s instructions [18]. Gene expression was analyzed by quantitative real-time polymerase chain reaction using the SYBERgreen master mix system according to the manufacturer’s protocol. As a control of the amplification specificity, a melting curve analysis was performed for each PCR experiment/each primers pairs. Relative quantification was determined using a standard curve made from a purified PCR product for each gene tested, with concentrations ranging from 10^−3^ to 10^−6^ ng/μl. For standardization of gene expression levels, results were expressed as the ratio of the mRNA level of each gene of interest versus the *RPS29* gene. This gene (*RPS29*), referred to as a housekeeping gene, is typically a constitutive gene that is expressed at relatively constant levels in all cells independent of experimental conditions [17, 19], here in TGF-β1-treatment MSCs, compared to control ITS culture conditions. Each measurement was performed at least three times in three different patients.

The chondrogenic genes were type II collagen (*COL2A1*; NM_001844) and its isoform *COL2B* (NM_033150.2), aggrecan (*ACAN*; NM_001135), *COMP* (NM_000095), SRY (sex-determining region Y)-box 9 (*SOX9*; NM_000346), and type X collagen (*COL10A1*; NM_000493). The fibrotic genes were type I collagen (*COL1A1*; NM_000088.3) and versican (*VCAN*; NM_001164098). The osteogenic gene was runt-related transcription factor 2 (*RUNX2*; NM_001278478).

### Biochemical analysis of GAG in MSC-seeded sponges at D28

Sponge lysates after digestion for RT-PCR were used to determine GAG content. This colorimetric assay uses dimethyl methylene blue (DMB, Sigma) dye, according to Goldberg’s method [20]. The absorbance was assessed at 525 nm with a spectrophotometer (Dynatech) and compared to the standard curve generated from chondroitin sulfate isolated from shark cartilage.

### Histological analysis of MSC-seeded sponges at D28

After fixation with 4% PFA for 24 h at 4 °C, the sponges were dehydrated with increasing solutions of alcohol and embedded in paraffin. Sections at a thickness of 5 μm were stained with hematoxylin-erythrosin-saffron to visualize cell morphology and with Alcian blue to visualize proteoglycan content.

### Immunohistochemical analysis of MSC-seeded sponges at D28

The detection of type II collagen in cartilaginous TE substitutes was performed using a primary monoclonal antibody (6B3, Labvision) at a dilution of 1/100 and the LSAB+ kit (HRP, Dako) based on avidin-biotin techniques as previously described [21]. The sections were counterstained with hematoxylin at 1/5 for 1 min (RAL, France).

### Densitometric analysis of glycosaminoglycans and type II collagen using ImageJ

Histological sections stained with Alcian blue used to visualize proteoglycans or treated by immunohistochemistry to identify collagen II were digitized using a light microscope (DMD 108, Leica). The staining area percentages of Alcian blue and IHC markers for collagen type II were determined using the image analysis software ImageJ as previously described [18].

### Statistical analysis

Each experiment was performed in triplicate for three patients. For gene expression, the ratio of mRNA levels of each gene to RPS29 gene expression is expressed for each condition and MSC origin at D28. The results are presented as the mean ± standard error of the mean. Statistical analysis was performed with *t* tests to evaluate the effect of TGF-β1 versus ITS separately for each cell group. In a second step, a significant interaction was assessed between groups by two-way ANOVA with Bonferroni’s post hoc test. Asterisks represent significant differences versus control condition (ITS 1%) (*p < 0.05; *p* < 0.01; and ***p < 0.001). Hash signs represent a significant difference between cellular groups (^#^*p* < 0.05; *p* < 0.01; and ^###^*p* < 0.001). Statistical analysis was performed with GraphPad Prism® V8.

## Results

### Trilineage differentiation potential of BM-, SM-, and SF-MSCs

**Fig. 1:**
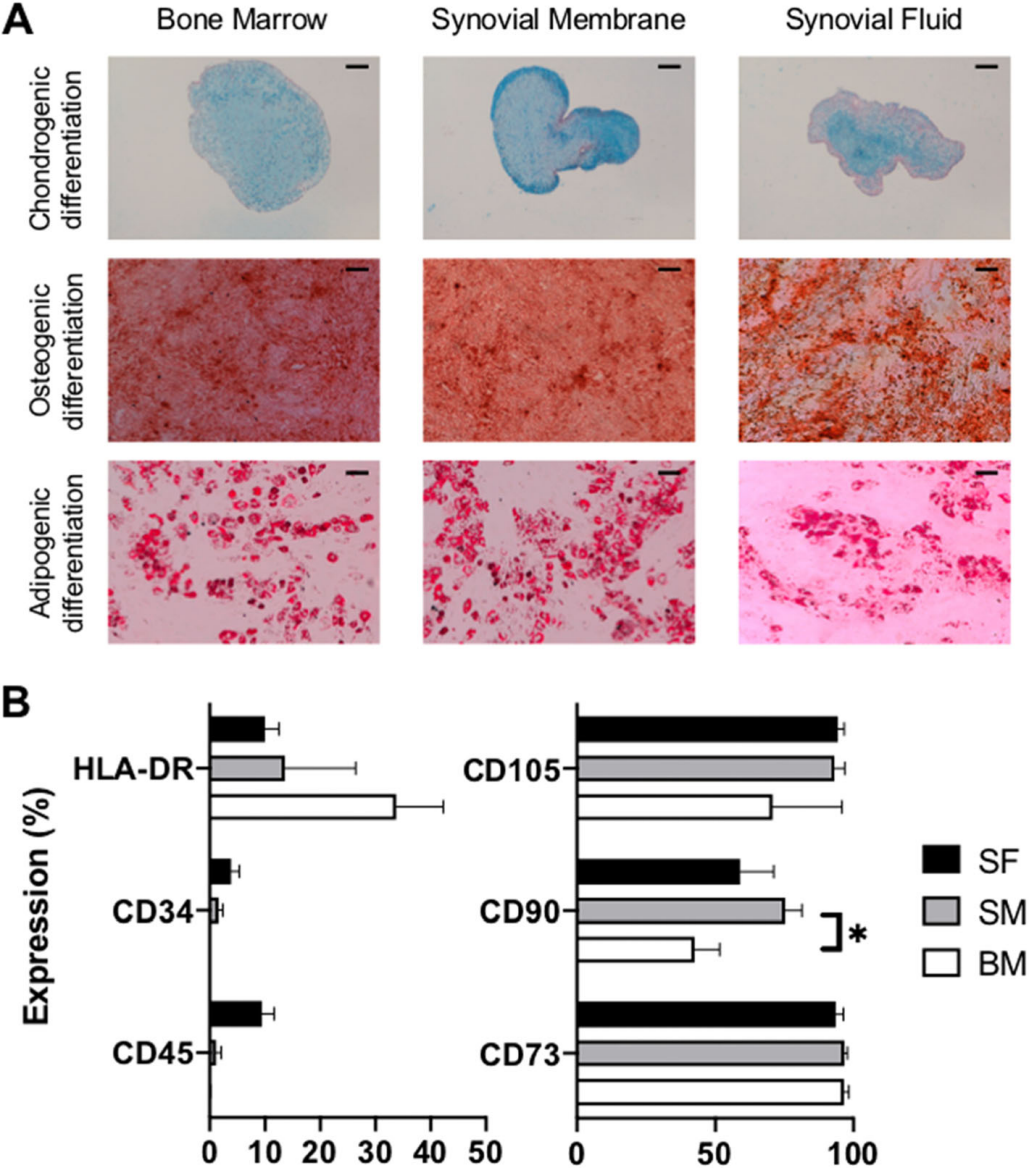
Trilineage differentiation and immunophenotyping of human SF-, SM-, and BM-MSCs. **a** Chondrogenic differentiation of MSCs was visualized by using Alcian blue staining of pellets cultured for 28 days in chondrogenic medium (TGF-β1). Osteogenic differentiation was induced in monolayer MSCs cultured for 14 days with osteogenic medium, and calcium deposits were highlighted using alizarin red staining. For adipogenic differentiation, MSCs were cultured in monolayers in specific medium for 21 days, and lipid droplets were observed using oil red staining. Scale bars = 200 μm. **b** Immunophenotyping of MSCs was assessed at the end of the monolayer second passage (P2) in the expansion medium. Data are presented as the mean of positive cells ± SEM. Please note that the percentage scale is not similar on both plots

At P2, BM-, SM-, and SF-MSCs cultured for 28 days in pellets in chondrogenic medium showed proteoglycan synthesis that was visualized by Alcian blue staining (Fig. 1a). Grossly, the BM-MSC pellets were larger and rounder than SM and SF pellets. SM-MSC pellets exhibited more intense Alcian blue staining than BM-and SF pellets. Likewise, the three lineages of MSCs were cultured in monolayers for 14 days with osteogenic medium, the presence of calcium deposits was visualized by using alizarin red staining, and the most intense staining was found in BM-MSCs. Furthermore, after 21 days of culture in the adipogenic medium, we detected lipid droplets, which are characteristic of adipogenic differentiation, by using red oil staining and found decreased intensity in SF pellets (PCR and macroscopic assessments available in Supplementary data 1 and 2).

### Immunophenotyping of BM-, SM-, and SF-MSCs

All three cellular groups exhibited phenotypic characteristics that were consistent with stemness at P2. Both of the hematopoietic markers CD45 (0.1%, 1.2%, and 9.4%, respectively, NS) and CD34 (0.02%, 1.6% and 3.9%, respectively, NS) were poorly expressed, despite a nonstatistical trend showing a slight increase in SF-MSCs. HLA-DR expression was quite similar between the three kinds of MSCs, despite a nonsignificant trend showing increased expression in BM-MSCs. CD73, CD90, and CD105 are mesenchymal markers. All MSCs expressed CD73 and CD105 (> 90%, NS) very strongly. CD90 expression was variable depending on the cell type; SM-MSCs had significantly stronger CD90 expression compared to that of BM-MSCs (Fig. 1b).

### Gene profiles at D28 of collagen sponges seeded with BM-, SM-, and SF-MSCs

#### Chondrogenic genes

**Fig. 2:**
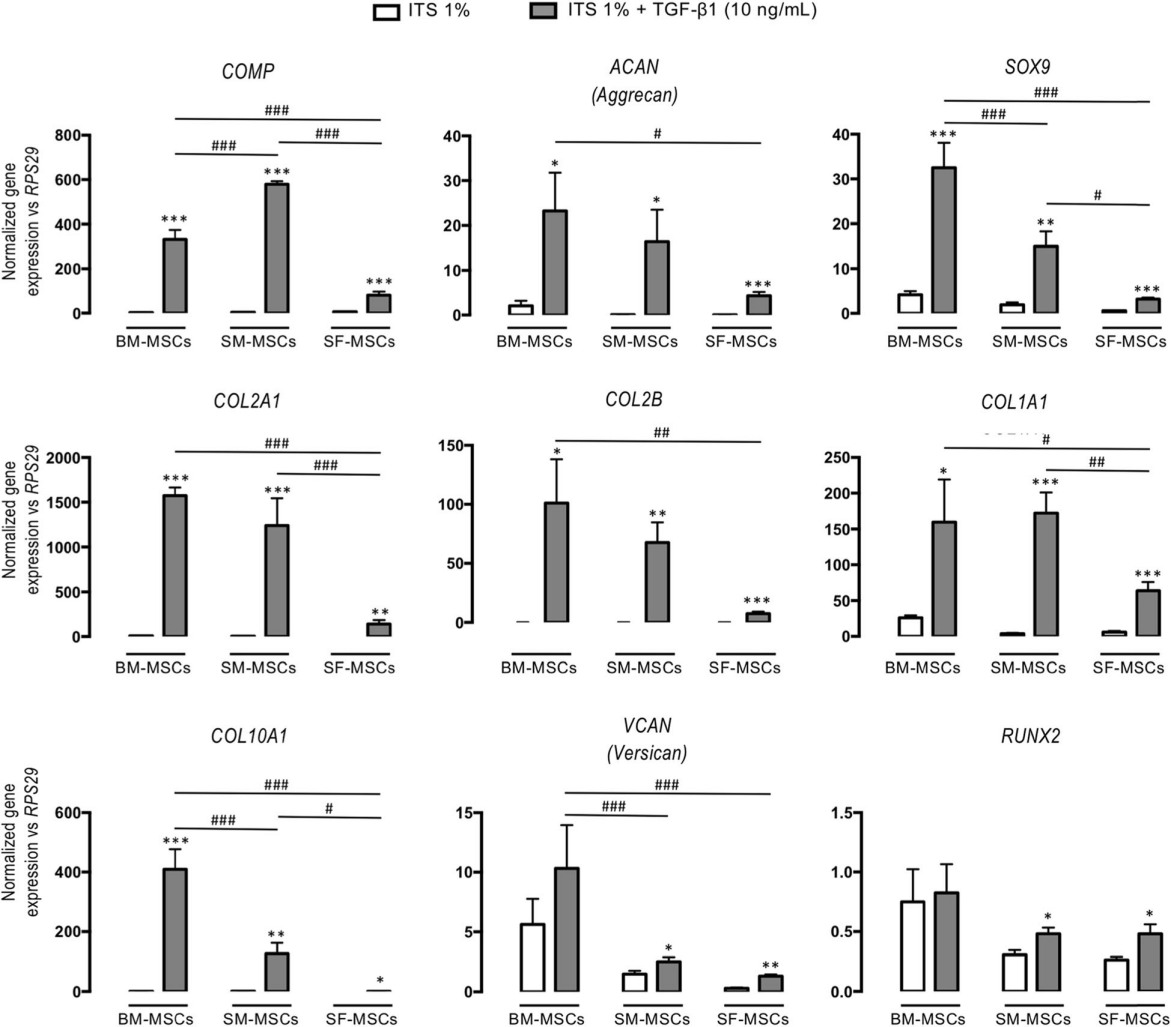
Gene expression at D28 in collagen sponges seeded with human BM-, SM-, and SF-MSCs under ITS and TGF-β1 conditions. Relative mRNA expression was measured by real-time polymerase chain reaction of chondrogenic (*COMP*, *ACAN*, *SOX9*, *COL2A1*, and *COL2B*), fibrotic (*COL1A1* and *VCAN*), and hypertrophic (*COL10A1* and *RUNX2*) markers. All results were normalized to RPS29 mRNA expression. Data are presented as the mean ± standard error of the mean. Statistical analysis was performed with a *t* test to evaluate the effect of TGF-β1 versus ITS for each cellular group separately and was followed by two-way ANOVA with Bonferroni’s post hoc test on all values to evaluate differences in gene expression between all cellular groups. Asterisks represent a significant difference versus control condition (ITS 1%); *p < 0.05, *p* < 0.01, ***p < 0.001. Hash signs represent a significant difference between cellular groups; ^#^*p* < 0.05, ^##^*p* < 0.01, ^###^*p* < 0.001

As depicted in Fig. 2, TGF-β1 strongly enhanced the expression of chondrogenic genes compared to that of ITS, especially in BM-MSCs and SM-MSCs. *COMP* expression was significantly increased in TGF-β1-treated SM-MSCs, but *ACAN*, *SOX9*, *COL2A1*, and the *COL2B* isoform expression was more pronounced in BM-MSCs compared with that of SM-MSCs. For all genes, SF-MSC expression was significantly low.

#### Fibrotic genes

Similarly, *COL1A1* overexpression was significantly increased by TGF-β1 equally in BM-MSCs and SM-MSCs. In addition, *VCAN* expression was increased in BM-MSCs. Again, TGF-β1-driven *COL1A1* and *VCAN* overexpression was significantly reduced in SF-MSCs.

#### Hypertrophic and osteogenic genes

*COL10A1*, a marker of hypertrophic differentiation, was strongly increased by TGF-β1, especially in BM-MSCs. SF-MSCs were less responsive than the other cells. *RUNX2* is a transcription factor that is involved in osteogenic differentiation, and its expression was very low in the three lineages, even under the influence of TGF-β1.

### GAG content in cartilage substitutes

**Fig. 3:**
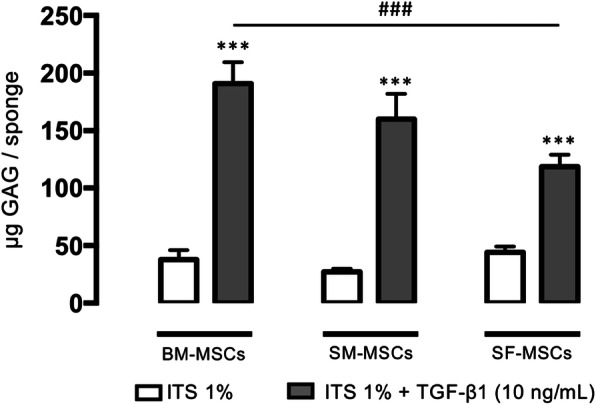
GAG content at D28 in collagen sponges seeded with advanced OA human BM-, SM-, and SF-MSCs under ITS and TGF-β1. The concentration of GAG in micrograms per sponge was measured with a dimethyl methylene blue colorimetric assay. Statistical analysis was performed with a *t* test to evaluate the effect of TGF-β1 versus ITS for each cellular group separately and followed by two-way ANOVA with Bonferroni’s post hoc test on all values to evaluate the difference in GAG production between all cellular groups. Asterisks represent a significant difference versus control condition (ITS 1%); ***p < 0.001. Hash signs represent a significant difference between cellular group; ^###^*p* < 0.001

The GAG content is shown in Fig. 3. For the three cell types, the 1% ITS control condition did not induce the production of GAG, while TGF-β1 (10 ng/mL) significantly induced synthesis in the three lineages. The average amount was 190 μg/sponge for BM-MSCs, 160 μg/mL for SM-MSCs, and 118 μg/mL for SF-MSCs, which was significantly lower than that of BM-MSCs.

### Histological and immunohistochemical analysis of cartilage substitutes

**Fig. 4:**
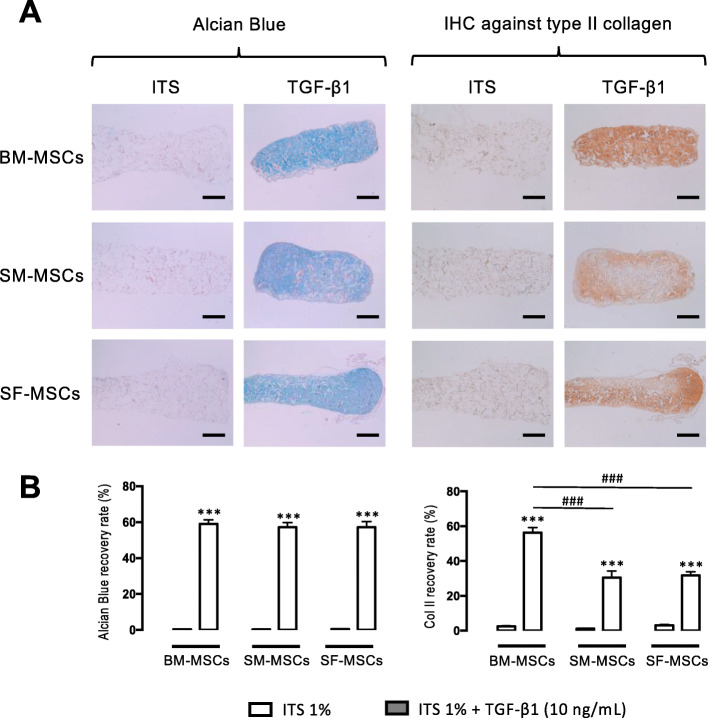
Histological and immunohistochemical analyses at D28 of collagen sponges seeded with advanced OA human BM-, SM-, and SF-MSCs under ITS and TGF-β1. **a** The proteoglycan content was visualized by Alcian blue staining, and type II collagen was highlighted by immunohistochemistry. The scale bars represent 400 μm. All observations were carried out on three different samples for each culture condition and for each patient. **b** For densitometry measurement of Alcian blue staining and type II collagen, statistical analysis was performed with a *t* test to evaluate the effect of TGF-β1 versus ITS for each cellular group separately and was followed by two-way ANOVA with Bonferroni’s post hoc test on all values to evaluate the difference in GAG production between all cellular groups. Asterisks represent a significant difference versus control condition (ITS 1%); ***p < 0.001. Hash signs represent a significant difference between cellular group; ^###^*p* < 0.001

HES revealed homogeneous distribution and no mortality in the cells within the sponge under both ITS and TGF-β1 conditions. As depicted in Fig. 4a, Alcian blue staining revealed no GAG synthesis at D28 under ITS conditions. In contrast, TGF-β1 significantly increased proteoglycan content in the three MSC-seeded substitutes without significant differences between the three cell lineages. Type II collagen labeling revealed that ITS failed to induce any matrix deposition, while TGF-β1 provoked significant type II collagen synthesis. The densitometry measurement (Fig. 4b) demonstrated significant differences between the three cell types; type II collagen content was significantly higher in BM-MSCs than in SM- and SF-MSCs. Under normoxic conditions, no calcium deposition was observed in the sponge of each cellular group (Supplementary data 3).

## Discussion

The main goal of this study was to directly compare the stemness, immunophenotype, and chondrogenic potential of human advanced OA BM-MSCs, SM-MSCs, and SF-MSCs differentiated in collagen sponges. To date, such a direct comparison has not been reported in humans. First, we confirmed that these three lineages were able to differentiate into chondrocytes, osteocytes, and adipocytes with dedicated media [22–24]. We also observed that MSCs derived from bone marrow, synovial tissue, and synovial fluid exerted a similar immunophenotype as demonstrated by cell marker expression [25]. Despite some variability, CD105 (endoglin), a stemness marker, was very highly expressed by the three MSC lineages. This transmembrane protein is involved in the TGF-β signaling pathways. Although debated [26], it is also an excellent predictor of MSC chondrogenic differentiation. For example, it is used to select SM-MSCs [27]. In addition, our results showed strong expression of CD73 in all three cell types. This enzyme that metabolizes AMP to adenosine is described as a marker that is associated with chondrogenic differentiation, and its expression decreases significantly with chondrogenic differentiation [28].

Interestingly, the stemness marker CD90 was less positive in BM-MSCs (approximately 50%) than in SM-MSCs (approximately 75%). With regard to the CD90/Thy-1 marker, it has been shown that this marker is expressed on the MSC surface, particularly on undifferentiated MSCs [28]. Moreover, its expression decreases once the MSCs engage in chondrogenic or osteogenic differentiation [29]. In physiological conditions, it has been shown that chondrocytes do not express CD90, and this expression increases during dedifferentiation in monolayers [30], thus confirming the association of marker expression with the undifferentiated status of our MSCs. Additionally, during the expansion stages, our medium contained 1 ng/mL of bFGF, which is known to induce cell proliferation. This supplementation of bFGF can modulate the expression of cell surface markers such as CD90 in SM-MSCs [31].

The negative markers CD34 and CD45 were poorly expressed. CD34 is expressed on the surface of hematopoietic stem cells (HSCs), and CD45 is present on all nucleated hematopoietic cells (except erythrocytes and platelets). Our results confirmed that HSC markers are not expressed on BM-, SM-, and SF-MSCs at P2. Interestingly, HLA-DR, a negative marker, was moderately expressed without a significant difference between the three MSC types. These results were surprising because HLA-DR is a surface receptor of the major histocompatibility complex that is generally associated with GvHD (graft versus host disease). It has been shown previously that BM-MSCs from healthy bone marrow express HLA-DR only after stimulation by IFN-γ [32]. In another study, the heterogeneity of HLA-DR expression in human BM-MSCs was reported [33]. Again, the use of bFGF in cellular amplification may also induce the expression of HLA-DR [34]. Finally, it has been shown that the immunophenotype of BM-MSCs can be altered by in vitro proliferation [35].

Inter-individual variability is likely to contribute to the intergroup variability of immunophenotypes [36]. All of the cells used here came from elderly patients with advanced osteoarthritis of the hip or knee. Thus, the age of the donor, drug treatments, and the pathological microenvironment may interfere. Anatomical differences may be of importance. BM-MSCs are not in contact with the joint cavity, while SM-MSCs are. In addition, SF-MSCs are immersed in hypoxic and acidic joint fluid [18] and are influenced by the inflammatory environment [37–39], and the resident senescent cells develop a detrimental paracrine senescence-associated secretory phenotype [40]. In contrast, in our experience, this immunophenotypic heterogeneity was not found in healthy neonatal MSC populations, such as MSCs derived from human umbilical cord blood or Wharton jelly [21].

To compare the respective BM-, SM-, and SF-MSC chondrogenic potential, we chose to use a porous support biomaterial based on type I collagen sponges that allows the synthesis of extracellular matrix within this biomaterial, which is unlike the pellet model, a 3D structure based on cell-cell interactions. Collagen sponges are useful for their compatibility with clinical practice and cartilage engineering strategies [41, 42]. This biomaterial allows attachment of the cells and the expression of chondrogenic markers when the cells are seeded in the presence of TGF-β1. This collagen scaffold is biodegradable and is made with type I atelocollagen that is not immunogenic. This collagen isotype is chemically crosslinked to obtain a 3D scaffold with excellent mechanical and thermal stability [43]. With this in mind, we confirmed that TGF-β1 strongly induced chondrogenesis in BM-MSCs [44], SM-MSCs [45, 46], and SF-MSCs [18, 36, 47]. These results were consistent with our previous work showing that the use of TGF-β1 induced strong overexpression of the genes of interest, such as type 2 collagen and aggrecan, after 28 days of BM-MSC differentiation in collagen sponges [48]. Additionally, our preliminary data confirmed that BMP-2 alone, a promoter rather than an inducer [9], did not promote chondrogenesis in these three MSCs lineages embedded in collagen sponges. Besides, we observed that TGF-β1, TGF-β3, TGF-β1+BMP-2 and TGF-β3+BMP2 were quite equipotent in terms of chondrogenesis at both genic and protein levels (unpublished internal data).

We also evaluated the expression of chondrogenic genes in MSC-seeded sponges by quantitative RT-PCR. As expected, ITS control conditions did not induce the expression of any genes of interest. In contrast, as previously reported [12], TGF-β1 induced, to varying degrees, the chondrogenic differentiation of MSCs from bone marrow, synovial membrane, and synovial fluid. These results are consistent with our previous work showing that the use of TGF-β1 induced strong overexpression of chondrogenic genes such as *ACAN*, *COL2A1*, and its isoform *COL2B* at D28 in MSC-seeded collagen sponges [48]. Interestingly, overexpression of these genes was higher in BM-MSCs and SM-MSCs than in SF-MSCs.

We also studied the expression of the transcription factors *SOX9* (the “*chondromaster*” gene) and *RUNX2* (the “*osteomaster*” gene). For all three cell types, *SOX9* expression was significantly increased in a similar manner, with BM-MSCs exerting the highest ratio and SF-MSCs the lowest. Conversely, *RUNX2* expression remained very low, suggesting that chondrogenic differentiation did not skew towards terminal ossification. Nevertheless, *COL10A1* was highly expressed in BM-MSCs, mildly in SM-MSCs, and poorly in SF-MSCs. This result is consistent with the literature because, under normoxia, chondrogenic differentiation is often associated with an increase in markers associated with hypertrophy [49–51]. These results fit with biochemical, histological, and immunohistochemical analyses. These invasive techniques provide qualitative information on cell distribution and the amount of neo-synthesized extracellular matrix (ECM). GAG content is less important for SF-MSCs. Interestingly, type II collagen (specific immunohistochemistry) was more abundant in BM-MSCs. It is important to note that the different cartilage substitutes did not show any calcification after 28 days, whereas calcification is classically observed with MSC-seeded alginate 3D culture under normoxia [52].

Several studies have compared the differentiation capabilities and chondrogenic potential of different sources of synovial stem cells from humans [53–56], horses [36, 57–59], dogs [60], and rats [61] versus bone marrow MSCs. Their results often showed superior chondrogenicity of cells derived from synovium (SM and/or SF) compared to that of BM-MSCs [62]. However, these studies were conducted in different species with various anatomical origins (hip, knee, and femoral diaphysis) and pathological conditions (temporomandibular joint dysfunction and anterior cruciate ligament rupture). Intraindividual variation in SF-MSC-related chondrogenicity between the hip and knee in patients who underwent both hip and knee arthroscopy on the same day has recently been demonstrated [63]. Furthermore, the three-dimensional differentiation system and growth factors were variable within the literature. Our study was conducted in patients with old age who were suffering from advanced OA, which may promote age-related alterations affecting the chondrogenicity of synovial MSCs, as recently demonstrated in an equine model [64] and in the clinic [65]. Additionally, changes in gene expression do not always correlate with protein synthesis, which may contribute to the differences observed in the 3 MSC lineages [66]. Apart from this in vivo senescence, whose influence on chondrogenicity remains debated [67], an in vitro replicative senescence (aging) might influence the results: environmental factors like enzyme-dependent cell detaching methods [68] or numbers of passages [69, 70] during expansion. These results are consistent with our internal data (Supplementary data 4). Interestingly, a robust work performed with BM-MSCs demonstrates that chondrogenicity is altered only after P6, osteogenic commitment after P5, while adipogenicity persists at P11. Furthermore, environmental conditioning, notably with FGF, rejuvenates MSCs’ proliferation and chondrogenic potential [71]. Finally, our chondrogenic gradient BM-MSCs > SM-MSCs > SF-MSCs is consistent with a previous study performed in similar patients on P3 with the pellet’s conditions under the influence of a mix of TGF-β3 + BMP-6 [72].

## Conclusion

We demonstrated that BM-, SM-, and SF-MSCs from advanced OA patients undergoing hip or knee arthroplasty are valuable candidates for cartilage engineering, although they show different immunophenotyping and chondrogenic properties. No studies have been conducted in the clinic comparing these three MSC lineages for cartilage engineering. All cell types were able to grow in monolayers and differentiate into adipogenic, osteogenic, and chondrogenic linages. The cells also responded to various levels of TGF-β1-driven 3D chondrogenic differentiation in collagen sponges. However, there was considerable variability within and between the lineages. Most likely, these differences were due to the detrimental priming of the OA-related intra-articular inflammatory environment on SM- and SF-MSCs; bone marrow MSCs probably remain the best candidate for hyaline cartilage engineering. In contrast, SF-MSCs did not overexpress hypertrophic genes under our currently described culture conditions.

## Supplementary information

**Additional file 1.** RT-PCR analysis of MSCs differentiation

**Additional file 2.** Osteogenic differentiation of MSCs issued from human bone marrow (BM-MSCs), from the human synovial membrane (SM-MSCs) and fluid (SF-MSCs) at D14 without (OSTEO-) or with osteogenic medium (OSTEO+). Adipogenic differentiation of MSCs issued from human bone marrow (BM-MSCs), synovial membrane (SM-MSCs) and synovial fluid (SF-MSCs) at D21 without (ADIPO-) or with adipogenic medium (ADIPO+)

**Additional file 3.** Von Kossa staining at D28 of collagen sponges seeded with advanced OA human BM, SM- and SF-MSCs under ITS and TGF-β1

**Additional file 4.** The senescence was evaluated using a kit "senescence β galactosidase staining kit" according to the manufacturer's recommendations (Cell Signaling Technology)

## Data Availability

The datasets taken during and/or analyzed during the current study are available from the corresponding author on reasonable request.

## Abbreviations

*ACAN*: Aggrecan (gene)
ACI: Autologous chondrocyte implantation
ANOVA: Analysis of variance
bFGF: Basic fibroblast growth factor
*BGLAP*: Osteocalcin (gene)
BM: Bone marrow
BMP-2: Bone morphogenetic protein 2
CO_2_: Carbon dioxide
*COL10A1*: Collagen type X, alpha 1 chain (gene)
*COL2A*: Collagen type II alpha 1 chain (gene)
*Col2b*: Collagen type II isoform (gene)
*COMP*: Cartilage oligomeric matrix protein (gene)
DMEM: Dulbecco’s modified Eagle medium
DNA: Deoxyribonucleic acid
ECM: Extracellular matrix
EDTA: Ethylene diamine tetra acetic acid
FBS: Fetal bovine serum
GAG: Glycosaminoglycan
GvHD: Graft versus host disease
HES: Hematoxylin erythrosine saffron
HSC: Hematopoietic stem cells
IHC: Immunohistochemistry
ITS: Insulin-transferrin-selenium
MSCs: Mesenchymal stromal cells
MTT: 3 (4,5-Dimethylthiazol-2-yl)-2,5-diphenyltetrazolium bromide
OA: Osteoarthritis
*OSX*: Osterix (gene)
P: Passage 1, 2, 3
PAD: Proline, L-ascorbic acid-2-phosphate and dexamethasone
PBS: Phosphate-buffered saline
PFA: Paraformaldehyde
qRT-PCR: Quantitative reverse transcription-polymerase chain reaction
RNA: Ribonucleic acid
*RPS29*: Ribosomal Protein S29 (gene)
SF: Synovial fluid
SM: Synovial membrane
*SOX9*: Sex-determining region-related HMG-box 9 (gene)
TGF-β1: Transforming growth factor beta 1
*VCAN*: Versican (gene)
