# Supplementary material for: Respective stemness and chondrogenic potential of mesenchymal stem cells isolated from human bone marrow, synovial membrane, and synovial fluid
Source: Stem Cell Res Ther. 2020 Jul 25;11:316. doi: 10.1186/s13287-020-01786-5 (PMC7382063; doi:10.1186/s13287-020-01786-5)
Supplement: Supplementary file 1 — Additional file 1. RT-PCR analysis of MSCs differentiation [file 13287_2020_1786_MOESM1_ESM.docx]

# Supplementary data 1


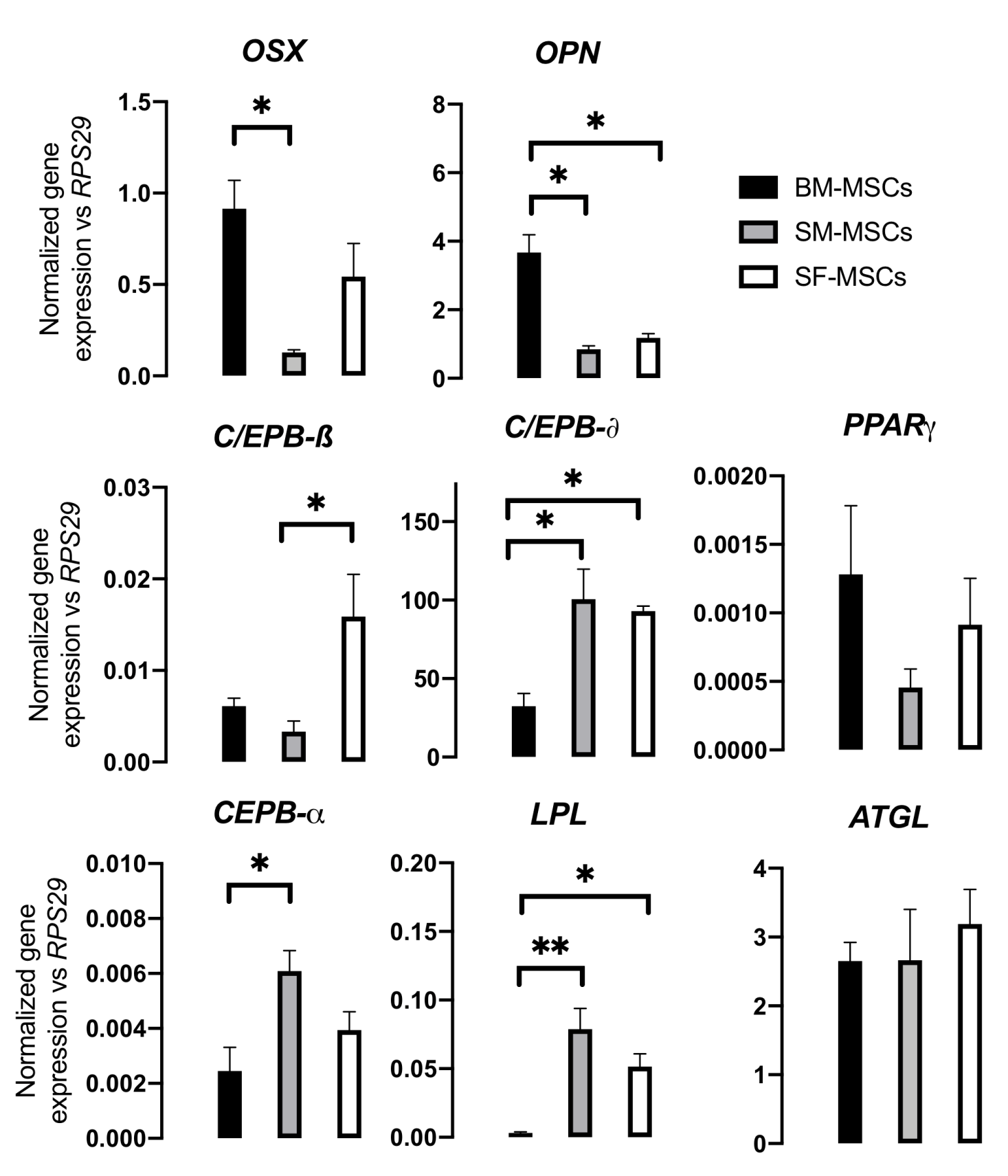


**RTPCR analysis of MSCs differentiation.**

Osteogenic differentiation was evaluated by determining the expression levels of osteopontin (OPN) and osterix (OSX), both involved in osteogenesis. Adipogenic differentiation was evaluated by determining the expression of genes involved in adipogenesis, such as C/EBPß and C/EBP∂ (early genes) and PPARγ, C/EBPα, LPL and ATGL (late genes). mRNA levels were normalized with respect to RPS29, which was chosen as an internal control (see Material and Methods). Each experiment was repeated three times. (mean ± SEM, **p* < .05, ** p<.001, ANOVA with Bonferroni’s post hoc test)

*See Methodology* in Di Bernardo G, Messina G, Capasso S, et al. Sera of overweight people promote in vitro adipocyte differentiation of bone marrow stromal cells. *Stem Cell Res Ther*. 2014;5(1):4. Published 2014 Jan 9. doi:10.1186/scrt393
