# Supplementary material for: Respective stemness and chondrogenic potential of mesenchymal stem cells isolated from human bone marrow, synovial membrane, and synovial fluid
Source: Stem Cell Res Ther. 2020 Jul 25;11:316. doi: 10.1186/s13287-020-01786-5 (PMC7382063; doi:10.1186/s13287-020-01786-5)
Supplement: Supplementary file 2 — Additional file 2. Osteogenic differentiation of MSCs issued from human bone marrow (BM-MSCs), from the human synovial membrane (SM-MSCs) and fluid (SF-MSCs) at D14 without (OSTEO-) or with osteogenic medium (OSTEO+). Adipogenic differentiation of MSCs issued from human bone marrow (BM-MSCs), synovial membrane (SM-MSCs) and synovial fluid (SF-MSCs) at D21 without (ADIPO-) or with adipogenic medium (ADIPO+) [file 13287_2020_1786_MOESM2_ESM.docx]

# Supplementary data 2


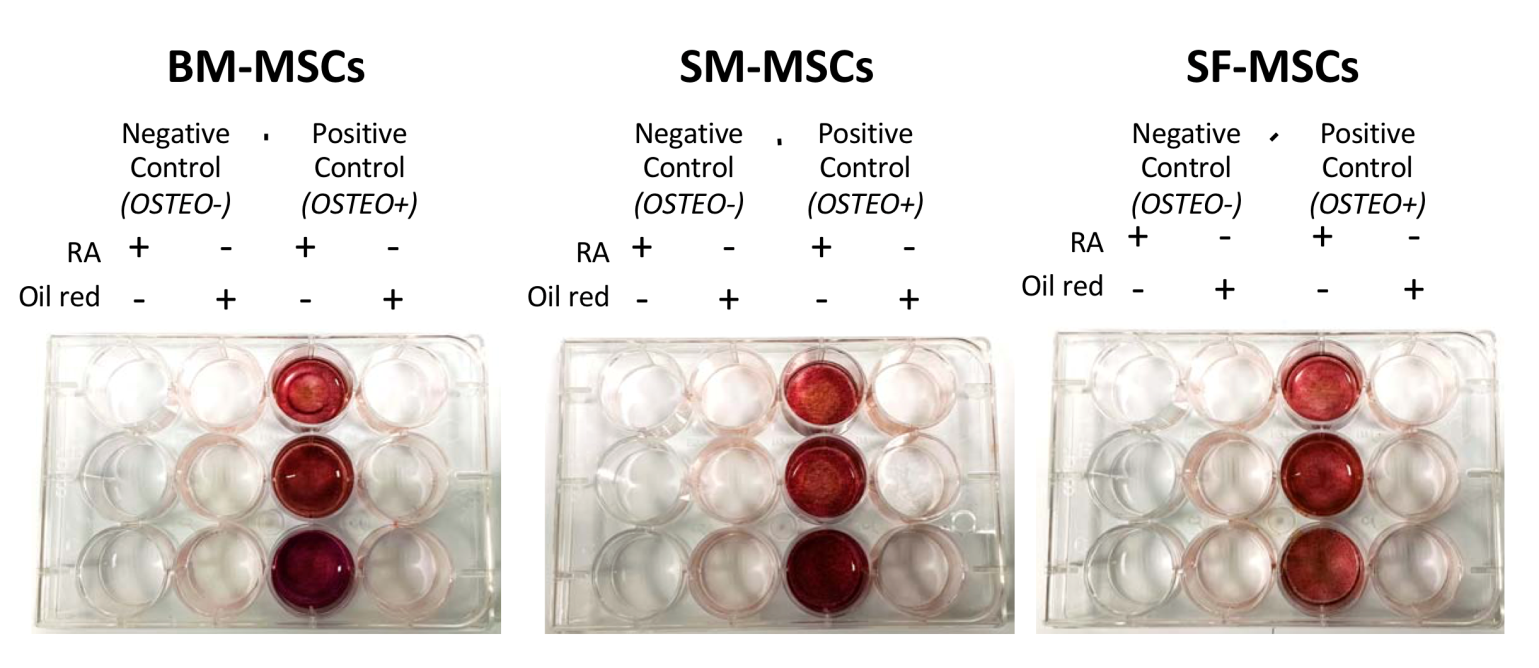


**Osteogenic differentiation of MSCs issued from human bone marrow (BM-MSCs), from the human synovial membrane (SM-MSCs) and fluid (SF-MSCs) at D14 without (OSTEO-) or with osteogenic medium (OSTEO+).**

For negative control, MSCs were cultured without an osteogenic medium. In the first column of culture plate, MSCs were stained with Alizarin Red (RA) to verify the absence of calcium deposits and in the second column with Oil red to verify the absence of lipid droplets. For positive control, MSCs were cultured with osteogenic medium and stained with alizarin red in the third column and with Oil red in the fourth column. We observed no adipogenic differentiation of MSCs with osteogenic medium. On the other hand, osteogenic differentiation was evident with the three sources of studied MSCs.


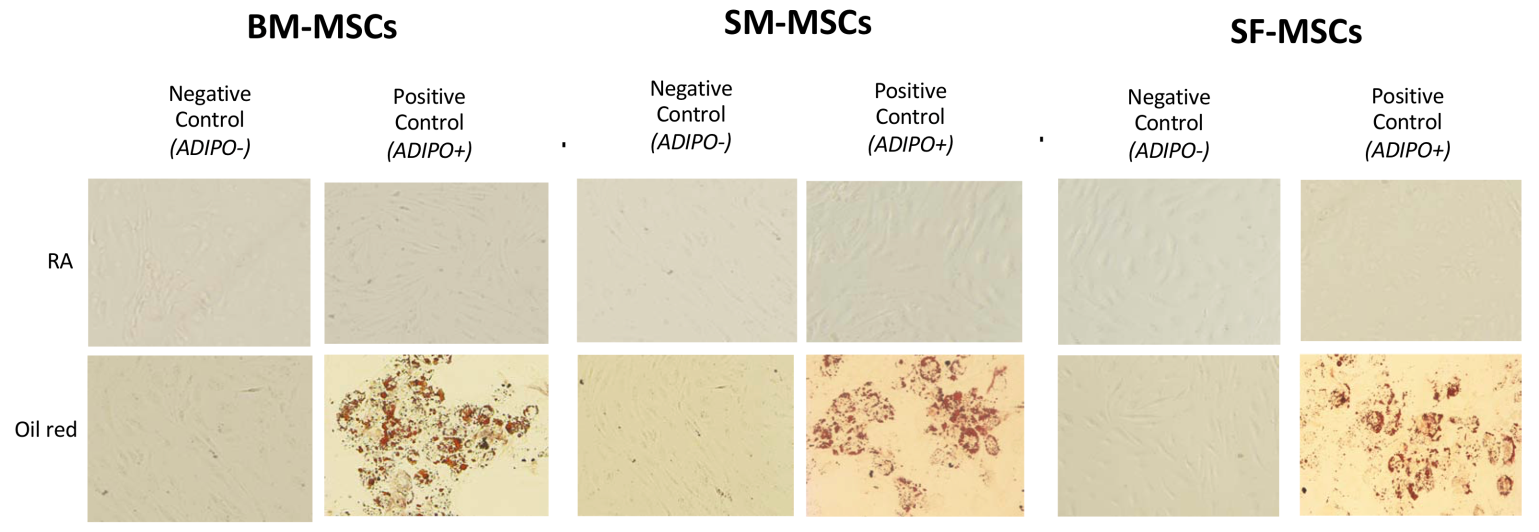


**Adipogenic differentiation of MSCs issued from human bone marrow (BM-MSCs), synovial membrane (SM-MSCs) and synovial fluid (SF-MSCs) at D21 without (ADIPO-) or with adipogenic medium (ADIPO+).**

For negative control, MSCs were cultured without an adipogenic medium. For negative control, MSCs were stained with Alizarin Red (RA) to verify the absence of calcium deposits and with Oil red to verify the absence of lipid droplets. For positive control, MSCs were cultured with adipogenic medium and stained with alizarin red and with Oil. We observed no osteogenic differentiation of MSCs with the adipogenic medium. On the other hand, an adipogenic differentiation was obvious with the three sources of studied MSCs.
