# Supplementary material for: Respective stemness and chondrogenic potential of mesenchymal stem cells isolated from human bone marrow, synovial membrane, and synovial fluid
Source: Stem Cell Res Ther. 2020 Jul 25;11:316. doi: 10.1186/s13287-020-01786-5 (PMC7382063; doi:10.1186/s13287-020-01786-5)
Supplement: Supplementary file 3 — Additional file 3. Von Kossa staining at D28 of collagen sponges seeded with advanced OA human BM, SM- and SF-MSCs under ITS and TGF-β1 [file 13287_2020_1786_MOESM3_ESM.docx]

# Supplementary data 3

**Von Kossa staining at D28 of collagen sponges seeded with advanced OA human BM, SM- and SF-MSCs under ITS and TGF-β1:**

Von Kossa stain is widely used in histology to detect the presence of abnormal calcium deposits. The principle of this coloration is based on the transformation of calcium salts into silver salts: calcium ions, bound to phosphates, are replaced by silver ions brought by a solution of silver nitrate. Placed under a light source, the silver phosphates undergo a photochemical degradation, leading to the observation of metallic silver deposits. Original magnification x 4. No calcification is detected in various conditions.
