# Supplementary material for: Respective stemness and chondrogenic potential of mesenchymal stem cells isolated from human bone marrow, synovial membrane, and synovial fluid
Source: Stem Cell Res Ther. 2020 Jul 25;11:316. doi: 10.1186/s13287-020-01786-5 (PMC7382063; doi:10.1186/s13287-020-01786-5)
Supplement: Supplementary file 4 — Additional file 4. The senescence was evaluated using a kit "senescence β galactosidase staining kit" according to the manufacturer's recommendations (Cell Signaling Technology) [file 13287_2020_1786_MOESM4_ESM.docx]

# Supplementary data 4


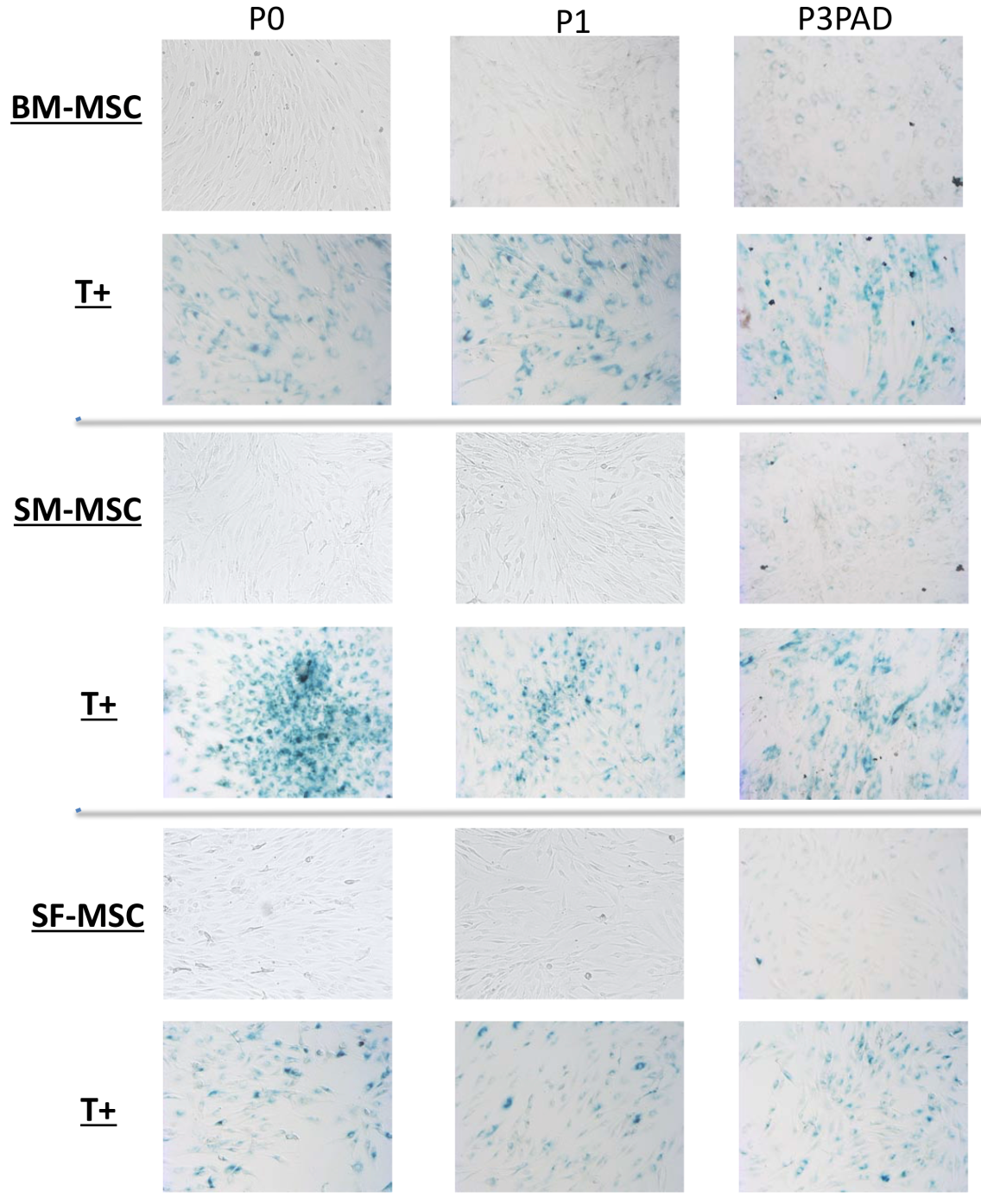


***The*senescence was evaluated using a kit "senescence β galactosidase staining kit" according to the manufacturer's recommendations (Cell Signaling Technology).**

For each type of cell, BM-, SM-, and SF- MSCs, three different patients were studied at P0, P1, and P3 PAD. For positive control, treatment with H2O2 (T+ 1 µl for 100 mL) was realized 24 hours before the test. Cells were then rinsed with 1X PBS and were fixed with the 1X Fixative Solution for 15 min at room temperature. β galactosidase staining solution was added, and the cells were then incubated overnight in a dry incubator. The blue color was finally observed under a microscope*. According to the manufacturer's recommendations, the senescence was evaluated using a kit "senescence β galactosidase staining kit" according to the manufacturer's recommendations (Cell Signaling Technology). For each type of cell, BM-, SM-, and SF- MSCs, three different patients were studied at P0, P1, and P3 PAD. For positive control, treatment with H2O2 (T+ 1 µl for 100 mL) was realized 24 hours before the test. Cells were then rinsed with 1X PBS and were fixed with the 1X Fixative Solution for 15 min at room temperature. β galactosidase staining solution was added, and the cells were then incubated overnight in a dry incubator. The blue color was finally observed under a microscope.*
